# Inhibitory effect of streptococci on the growth of *M. catarrhalis* strains and the diversity of putative bacteriocin-like gene loci in the genomes of *S. pneumoniae* and its relatives

**DOI:** 10.1186/s13568-017-0521-z

**Published:** 2017-12-13

**Authors:** L. N. Ikryannikova, M. V. Malakhova, G. G. Lominadze, I. Yu. Karpova, E. S. Kostryukova, N. A. Mayansky, A. N. Kruglov, E. A. Klimova, E. S. Lisitsina, E. N. Ilina, V. M. Govorun

**Affiliations:** 1 Federal Research and Clinical Center of Physical-Chemical Medicine, Malaya Pirogovskaya str., 1a, Moscow, 119435 Russia; 2 Federal State Budgetary Inst. “Scientific Center of Children Health” of RAMS, Moscow, Russia; 3 I.M. Sechenov First Moscow State Medical University, Moscow, Russia; 4 National Agency for Clinical Pharmacology and Pharmacy, Moscow, Russia; 5 A.I. Evdokimov, Moscow State University of Medicine and Dentistry, Moscow, Russia; 6 LTD Scientific and Industrial Company “Lytech”, Moscow, Russia

**Keywords:** Viridans group streptococci, *S. pneumoniae* and its relatives, *M. catarrhalis* growth inhibition, Bacteriocin-associated gene loci

## Abstract

*S. pneumoniae* is a facultative human pathogen causing a wide range of infections including the life-threatening pneumoniae or meningitis. It colonizes nasopharynx as well as its closest phylogenetic relatives *S. pseudopneumoniae* and *S. mitis*. Both the latter, despite the considerable morphological and phenotypic similarity with the pneumococcus, are considerably less pathogenic for humans and cause infections mainly in the immunocompromized hosts. In this work, we compared the inhibitory effect of *S. pneumoniae* and its relatives on the growth of *Moraxella catarrhalis* strains using the culture-based antagonistic test. We observed that the inhibitory effect of *S. mitis* strains is kept when a hydrogen peroxide produced by cells is inactivated by catalase, and even when the live cells are killed in chloroform vapors, in contrast to the pneumococcus whose inhibiting ability disappeared when the cells die. It was suggested that this effect may be due to the production of bacterial antimicrobial peptides by *S. mitis*, so we examined the genomes of our strains for the presence of bacteriocin-like peptides encoding genes. We observed that a set of bacteriocin-like genes in the genome of *S. mitis* is greatly poorer in comparison with *S. pneumoniae* one; moreover, in one *S. mitis* strain we found no bacteriocin-like genes. It could mean that there are probably some additional opportunities of *S. mitis* to inhibit the growth of competing neighbors which are still have to be discovered.

**Electronic supplementary material:**

The online version of this article (10.1186/s13568-017-0521-z) contains supplementary material, which is available to authorized users.

## Introduction

*S. pneumoniae* is a facultative pathogen causing a wide range of infections in children and adults, often with fatal outcome (van der Poll and Opal 2009; Donkor 2013). It colonizes human nasopharynx and can further migrate through the Eustachian tubes to cause otitis media, descend the respiratory tract to cause pneumonia or invade the bloodstream through the respiratory epithelium to cause bacteremia and meningitis, or spontaneously disappear over time (Shak et al. 2013). Along with *S. pneumoniae*, other streptococcal species including the closest phylogenetic relatives of pneumococci—*S. pseudopneumoniae* and *S. mitis*—are common residents of nasopharynx. Despite the considerable morphological and phenotypic similarity, which often prevents the correct identification and differentiation of these three species, they are very different in degrees of injuriousness to humans: while *S. pneumoniae* is associated with life-threatening diseases (pneumoniae or meningitis), *S. mitis* is a commensal causing infections mainly in the immunocompromized hosts (Teles et al. 2011; Mitchell 2011; Kilian et al. 2008). A special place belongs to *S. pseudopneumoniae*, whose pathogenic potential is still controversial (Harf-Monteil et al. 2006; Keith et al. 2006; Keith and Murdoch 2008; Fuursted et al. 2016). A lot of efforts were expended to explain the significant differences in pathogenicity between pneumococci and their genetic relatives. It was shown that the *S. mitis* genome contains homologues for many pneumococcal virulence factors involved in colonization and adherence including genes of surface proteins HtrA, ZmpB and PavA, choline-binding proteins LytB, LytC, CbpD and CbpE, lipoprotein SlrA etc., whereas some of them like the hyaluronidase gene *hysA*, *ply*, and *lytA* are absent (Denapaite et al. 2010). Also, in *S. mitis*, the virulence gene repertoire may vary from strain to strain. The current concept postulates that the full virulence factor arsenal is required to overcome the human immune defense as successfully as *S. pneumoniae* does (Mitchell 2011; Doern and Burnham 2010; Whatmore et al. 2000).

Pneumococcus and its relatives are not the only inhabitants of the nasopharynx. In accordance with the some investigations, more than 600 bacterial species can reside in the oral cavity—gateway into the upper respiratory tract (Dewhirst et al. 2010). The most common bacterial families besides Streptococcaceae were found Moraxellaceae, Corynebacteriaceae, Pasteurellaceae (including the genus *Haemophilus*) and Staphylococcaceae (Shak et al. 2013; Pettigrew et al. 2008), which colonize nasopharynx for the first months of life. Generally, a composition of the upper respiratory tract microbiome varies greatly among individuals and over time. It is influenced by many factors such as the host genetic background, age, social status, antibiotic use, vaccination, season, smoking etc. Among other factors, one of the most important is the interaction between microbes, including competitive one (Pettigrew et al. 2008; Bosch et al. 2013; Chen et al. 2015). In this work, we tried to evaluate and compare the competitive potential of *S. pneumoniae* and its closest commensal relatives (*S. pseudopneumoniae* and *S. mitis*) against *Moraxella catarrhalis* strains using both the traditional culture-based antagonistic tests and in silico searching of the genes encoding putative antimicrobial peptides across the genomes of the study strains. In our view, the results of this study can be valuable in terms of the competition inside the microbial community may impact nasopharyngeal dynamics and carriage of pathogenic or potential pathogenic bacteria. Therefore, an understanding of features of microbe–microbe interactions in the upper respiratory tract could provide not only the better insight into the pathogenesis of respiratory diseases, but maybe new tools to manage a microbial community for the human.

## Materials and methods

### Strains and their identification

Nine clinical isolates of viridans group streptococci were provided by the different clinical agencies of Moscow. Two *S. pneumoniae* isolates (Spn_357 and Spn_2009) were kindly provided by the A.I. Evdokimov Moscow State University of Medicine and Dentistry, and they were collected from the patients diagnosed with a sepsis or purulent meningitis. Two unencapsulated or non-typeable (NT) S*. pneumonia*e (Spn-NT_13856 and Spn-NT_2298) as well as two *S. pseudopneumoniae* (Spspn_G42 and Spspn_22725) isolates were acquired from the nasopharynx of paediatric patients of the Moscow Scientific Centre of Children Health, who were hospitalized with the different diagnosis, and three *S. mitis* isolates (Sm_11/5, Sm_13/39 and Sm_18/56) have been obtained from the Moscow National Agency for Clinical Pharmacology and Pharmacy. All isolates were routinely characterized by the standard viridans group streptococci identification tests under acquisition.

Being transferred to our laboratory, isolates were streaked out on the plates of Columbia agar (Oxoid Ltd., UK) supplied with a 5% of sheep blood, to form isolated single colonies; pure cultures were subcultured from single colonies after the overnight incubation at 37 °C in air with 5% CO_2_. All strains were re-tested: the optochin (OPT) susceptibility and bile solubility tests were made using the standard diagnostic optochin or sodium deoxycholate discs (Research Centrum on Pharmacotherapy, St. Petersburg, Russia) respectively, in accordance with the manufacturer’s instructions. The latex agglutination assay was accomplished by using of “Slidex® pneumo-kit” (bioMerieux®, France).

Main features of strains under study are presented in Table 1. Two strains, Spn_357 and Spn_2009, were “ordinary” pneumococci demonstrating the expected reactions for all routine identification tests. They were susceptible to OPT and sodium deoxycholate (“bile”), and agglutinated with the latex particles in “Slidex® pneumo-kit” assay. Multilocus sequence analysis (MLSA) attributed both isolates to the *S. pneumoniae* group. Two other *S. pneumoniae* strains, Spn-NT_13856 and Spn-NT_2298, were not “ordinary” pneumococci. These strains were unencapsulated, or non-typeable (NT), and they demonstrated negative reactions in latex agglutination and bile solubility tests; one strain, Spn-NT_13856, was resistant to OPT. Nevertheless, both strains appeared in the pneumococcal cluster by MLSA and had sequence type ST2996, as MLST procedure showed. This ST was firstly assigned to a strain selected in 2006 in Arkhangelsk (Russia) and belongs to a large clonal family of the NT pneumococci (http://pubmlst.org/spneumoniae). These interesting strains have been discussed in more details in (Ikryannikova et al. 2016).

**Table 1:** Characterization of study strains

Strain ID (VKM^a^ ID)	Species	Provider^b^/isolation year	Isolate source/patient age	Identification				
				OPT test (CO_2_ atm.)	Bile solubility	Latex agglutination test (“Slidex® pneumo-kit”)	Serotype^d^	Sequence type (MLST)
Spn_357 (VKM B-3128)	*S. pneumoniae*	EMSUMD/2008	Cerebrospinal fluid/adu	pos.	pos.	pos.	23F	ST 81
Spn_2009 (VKM B-3127)	*S. pneumoniae*	EMSUMD/2008	Blood/n.d.	pos.	pos.	pos.	22 F/A	ST 1470
Spn-NT_13856 (VKM B-3125)	*S. pneumoniae*	SCCH/2013	Nasopharynx/ped	neg.	neg.	neg.	NT^b^	ST 2996
Spn-NT_2298 (VKM B-3126)	*S. pneumoniae*	SCCH/2013	Nasopharynx/ped	pos.	neg.	neg.	NT	ST 2996
Spspn_G42 (VKM B-3123)	*S. pseudopneumoniae*	SCCH/2013	Nasopharynx/ped	neg.^c^	neg.	neg.	–	–
Spspn_22725 (VKM B-3124)	*S. pseudopneumoniae*	SCCH/2013	Nasopharynx/ped	neg.^c^	neg.	neg.	–	–
Sm_11/5 (VKM B-3130)	*S. mitis*	NACPP/2009	Nasopharynx/adu	neg.	neg.	neg.	–	–
Sm_13/39 (VKM B-3131)	*S. mitis*	NACPP/2009	Nasopharynx/ped	neg.	neg.	neg.	–	–
Sm_18/56 (VKM B-3129)	*S. mitis*	NACPP/2009	Nasopharynx/adu	neg.	neg.	neg.	–	–

*pos*. positive, *neg*. negative, *n.d.* no data, *adu* adult, *ped* pediatric, *NT* non-typeable

^a^VKM is all-Russian collection of microorganisms (http://www.vkm.ru/) in which all strains under study are deposited

^b^EMSUMD—A.I. Evdokimov Moscow State University of Medicine and Dentistry, Moscow, Russia; SCCH—Federal State Budgetary Inst. “Scientific Center of Children Health” of RAMS, Moscow, Russia; NACPP—National Agency for Clinical Pharmacology and Pharmacy, Moscow, Russia

^c^Some zones of inhibition (less than 14 mm) near OPT discs were observed for these strains under culturing in CO_2_ atmosphere, in contrast to culturing in air, where zones of inhibition were 18–20 mm and more

^d^Serotypes of pneumococcal strains were determined by inspection of the nucleotide sequences of genes coding the fragments of capsules, in accordance with the CDC recommendation (http://www.cdc.gov/streplab/protocols.html) (see “Materials and methods”)

Two *S. pseudopneumoniae* strains were initially attributed to the mitis group non-pneumococci by routine identification tests. However, in contrast to *S. mitis* strains, these pseudopneumococci reproducibly demonstrated clear 8–10 mm inhibition zones around the OPT discs being cultivated in 5% CO_2_ atmosphere. According to (A decree of the Ministry of Public Health of Russian Federation 1985), these strains should be referred to as OPT-nonsusceptible. However, in the ambient atmosphere, both strains were susceptible to OPT (inhibition zone > 14 mm). MLSA analysis unambiguously assigned these strains to the *S. pseudopneumoniae* cluster.

Strains have been kept in the laboratory strains bank at − 70 °C in Brain Heart Infusion broth (BD, USA) supplemented with 30% of fetal bovine serum (Gibco, USA) and 20% of glycerol. Also, all strains were deposited into the all-Russian collection of microorganisms (http://www.vkm.ru/) and available on request (see strains VKM identifiers in Table 1).

*Moraxella catarrhalis* strains (Mc51, Mc76 and Mc49) were provided from the LTD Scientific and Industrial Company “Lytech”.

### DNA extraction

For all genetic manipulations, total streptococcal DNA was extracted using the modified protocol of Miller et al. (1988). Briefly, 18 h culture from two blood agar plates was harvested and lysed in the Promega Nuclei Lysis Solution buffer (Promega, USA). After that, the cellular proteins were removed by adding of the saturated NaCl solution, and the genomic DNA was concentrated and desalted by isopropanol precipitation. Final DNA pellet was re-suspended in 50–100 μl of TE buffer and kept at 4 °C. For whole genome sequencing, DNA was additionally purified by using of minicolumns for DNA purification (“Technoclon”, Russia), in accordance with the manufacturer’s instructions.

### Genetic identification: MLST and MLSA schemes

Multilocus sequence typing and MLSA were performed as described by Enright and Spratt (1998) and by Bishop et al. (2009) respectively, with minor modifications for MLSA scheme described earlier in (Ikryannikova et al. 2011). Results were analyzed using the MLST (http://www.mlst.net) and MLSA (http://viridans.emlsa.net/) databases. Vector NTI 9.0 and MEGA 6.0 software was used for the manipulations with gene fragments and phylogenetic evolutionary analysis.

Multilocus sequence analysis and MLST gene fragments were repeatedly inspected when getting the whole genome nucleotide sequences of strains (see below). Looking for the MLSA or MLST genes in whole genome nucleotide sequences of the strains under study was realized using of the BLAST v. 2.2.23+ software.

### Assay for the growth inhibitors production

Screening for the production of inhibitory agents was based on a dual-layer agar plate technique. The bottom layer consisted of 10 ml of 1.5% LB broth agar (Amresco, USA) on which the inhibitor-producing test strain grew, and the top layer consisted of 6 ml of soft 0.7% agar (Helicon, Russia) containing 1% tryptone, 0.5% yeast extract (Oxoid Ltd., UK) and 0.1% NaCl, to support the growth of the indicator strain (*M. catarrhalis*). To screen for inhibitory effect, 18 h test strains grown on the Columbia blood agar (Oxoid Ltd., UK) plates were stabbed into the bottom LB broth agar layer and incubated for 18 h at 37 °C in air with 5% CO_2_. In some cases, LB broth agar surfaces were treated with catalase (4000 or 10,000 units per plate) before stabbing of the test strain. Indicator strains were cultured on the Columbia blood agar plates. Cells were harvested by the 10 µl microbiological loop and suspended in 300 µl of Brain Heart Infusion broth (BD, USA) to 1 MF, and then 100 µl of the indicator strain culture was added to the 6 ml of the soft agar and gently stirred. The bottom agar layer stabbed by the test strains was subsequently overlaid with a thin layer of a soft agar containing the indicator strain. Dual-layer plates were further incubated at 37 °C in air with 5% CO_2_. When necessary, the bottom agar layer supporting the growth of the test strains was kept in chloroform vapors for 15 min and then overlaid with a soft agar containing the indicator strain. Finally, zones of growth inhibition of indicator strain by test strains were inspected after the 10 h of growth.

### Whole genome sequencing and assembly

Whole genome nucleotide sequences of two strains (Spn-NT_2298 and Spn_22725) were obtained by using the Roche 454 Life Sciences Genome Sequencer FLX+ Genetic Analyzer (Roche 454 Life Science, USA), in accordance with the manufacturer’s instructions. Other strains were sequenced by using of the Ion Torrent PGM Genetic Analyzer (Life Technologies, USA). Details of sequencing are given in Additional file 1: Table S1. Genomes were assembled by GS De Novo Assembler v. 2.8 (Roche, USA). Assembly data were annotated using the RAST (Rapid Annotation using Subsystem Technology, USA http://rast.nmpdr.org/) and NCBI (American National Center for Biotechnology Information) PGAP (Prokaryotic Genome Annotation Pipeline, USA, http://www.ncbi.nlm.nih.gov/genome/annotation_prok/) Annotation Servers, and published in the GenBank database of the NCBI under Accession Numbers listed in Additional file 1: Table S1.

### Drawing of whole genome data for the analysis of the bacteriocin production associated clusters and getting of the additional information of strains

For the looking of genes encoding the potential bacteriocins in the genomes of strains under study, the open web-resource BAGEL3 (http://bagel.molgenrug.nl/index.php/bagel3) (de Jong et al. 2010) was used. BAGEL3 is one of the most significant world databases of bacteriocin-like peptides; this resource have a search tool allowing to identify putative bacteriocins on the basis of conserved domains, physical properties and the presence of biosynthesis, transport and immunity genes in their genomic context. Additionally, we have utilized the results of the investigations of Majchrzykiewicz (2011) whose work on the study of bacteriocin associated loci in *S. pneumoniae* genome seems to be most detailed.

Looking for the fragments of capsule operon in the genomes of the strains under study was performed using of the BLAST v. 2.2.23+ software. Investigation of the nucleotide sequences of genes coding the fragments of capsules was used to determine the serotypes of *S. pneumoniae* strains, in accordance with the CDC recommendation (http://www.cdc.gov/streplab/protocols.html).

## Results

### Inhibitory effect of streptococci on the growth of *Moraxella catarrhalis*

In the cases when the bottom agar layer stabbed with the test strains was not treated with catalase, we have observed very extensive, often merging zones of *M. catarrhalis* strains growth inhibition. Six test strains stabbed uniformly across the agar surface of 9-mm Petri dish completely inhibited the growth of indicator strains. Treatment of the agar surface with catalase (4000 or 10,000 units per plate) lead to a drastic decrease of the inhibition zones (Table 2). Catalase treatment followed by killing of live bacterial cells in chloroform vapors lead to the complete suppression of the inhibitory effects of pneumococci and pseudopneumococci, but no of *S. mitis* strains (Table 2).

**Table 2:** Inhibitory effect of streptococci under study on the growth of *M. catarrhalis* strains

	Size of zones of the inhibition of *M. catarrhalis* str. 51 growth by streptococci, mm^a^			
	+ catalase (4000 units/plate)	+ catalase (4000 units/plate) + chloroform	+ catalase (10,000 units/plate)	+ catalase (10,000 units/plate) + chloroform
Test strains				
*S. pneumoniae*				
Spn_357	5/6^b^	0/0	2	0
Spn_2009	9/10	0/0	10	0
*NT S. pneumoniae*				
Spn-NT_13856	3/8	0/0	2	0
Spn-NT_2298	8/5	0/0	4	0
*S. pseudopneumoniae*				
Spspn_G42	3/2	0/0	4	0
Spspn_22725	3/5	0/0	2	0
*S. mitis*				
Sm_11/5	2/5	9	5	7
Sm_13/39	3/5	5	4	6
Sm_18/56	7/12	6	7	7

^a^In the cases when the bottom agar layer stabbed with the test strains was not treated with catalase, we have observed very extensive, often merging zones of *M. catarrhalis* strains growth inhibition, or no growth of the indicator strain at all

^b^Slash marks the repeat of experiments

### Comparative screening of gene clusters associated with bacteriocin production in VGS strains of the mitis group

#### *blp* and *cibAB* loci

In accordance with the data collected for *S. pneumoniae* and its related species, there are at least two gene clusters associated with the bacteriocin production: *blp* (bacteriocin-like peptides; earlier *pnc* or *spi*) operon and *cibAB* (competence induced bacteriocins) cluster. Both are well described (see Ref. Lux et al. 2007; Son et al. 2011; Kjos et al. 2016; Miller et al. 2016; Bogaardt et al. 2015), although the confusion in names and the location of constituent genes, due to their complex structure, still occurs.

**Fig. 1:**
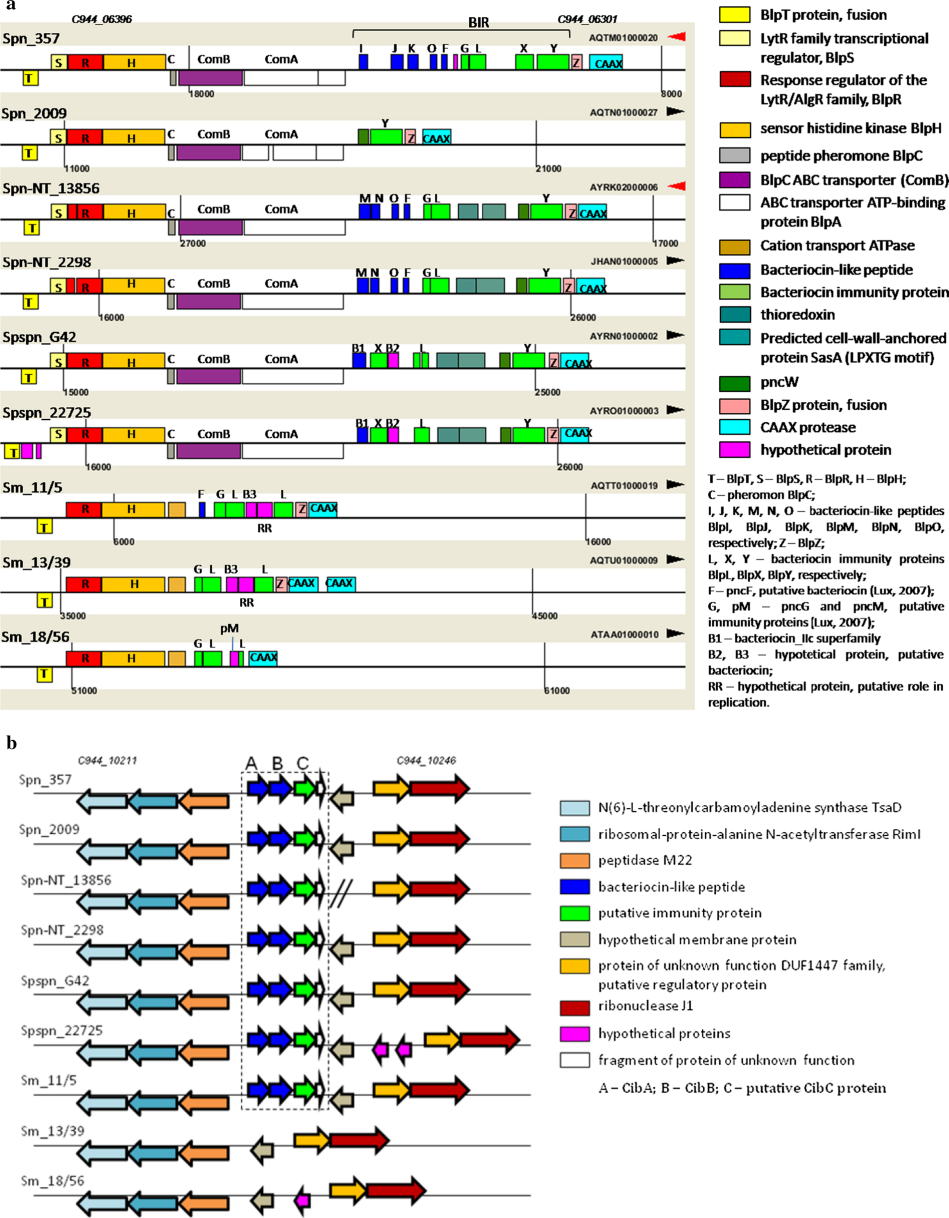
Reconstruction of *blp* (**a**) and *cibAB* (**b**) gene cluster structures according to genome analysis of nine VGS strains under study. The upper picture is prepared using of JContextExplorer v. 3.0 program (Seitzer et al. 2013). Homologous genes in compared samples are indicated by the same color (excluding the bacteriocin- and immune protein coding genes in the upper picture). Double slash in the lower picture indicates the gap in the nucleotide sequence of Spn-NT_13856 (a point of joining of two contigs). Here and further: NCBI identifiers of the first and last genes of genome fragments presented are given for Spn_357

The reconstruction of *blp* cluster in genomes of our strains is given in Fig. 1a. This locus was similar in all pneumococci and pseudopneumococci having an intact regulatory part and BIR consisting of a different number of bacteriocins- and immunity proteins coding genes. Spn_2009 was the exception missing almost all BIR (Bacteriocin Immunity Region) genes. Notably, comparing to *S. pneumoniae* strains, pseudopneumococci possessed a significantly smaller number of bacteriocin coding genes inside the BIR region.

In *S. mitis* genomes, *blp* cluster was also present, although it was substantially different from *S. pneumoniae* one. The *S. mitis blp* cluster conserved the regulatory *blpRH* system genes, while the *blpC* pheromone encoding gene as well as the ABC transporter genes were lost. Two of three *S. mitis* genomes contained no bacteriocin coding genes inside of the BIR region, although there were genes coding putative immune proteins.

Two-peptide class II CibAB bacteriocin is presumably the part of fratricidal killing pathway (Guiral et al. 2005). It was shown for *S. pneumoniae* that when becoming competent streptococcal cells produce a set of factors triggering the lysis of clonal but non-competent relatives. This mechanism is named fratricide, and CibAB bacteriocin was found to be one of the effectors of this process. *cibAB* cluster was presented in all pneumococcal and pseudopneumococcal genomes under study, but was not found in two of three *S. mitis* genomes (Fig. 1b). Note that the *cibC* gene downstream of *cibAB* was missed in the annotations and was deduced by analyses of the corresponding nucleotide sequences, based on the nucleotide sequence of *S. mitis* B6 *cibC* gene (smi_1957). Thus, the *cibAB* bacteriocin cluster did not appear to be specific for *S. pneumoniae,* opposing an earlier suggestion (Majchrzykiewicz 2011).

Our next step was to search for genes coding potential bacteriocin peptides in the genomes of strains under study using BAGEL3 web-resource.

#### Lantibiotic clusters

**Fig. 2:**
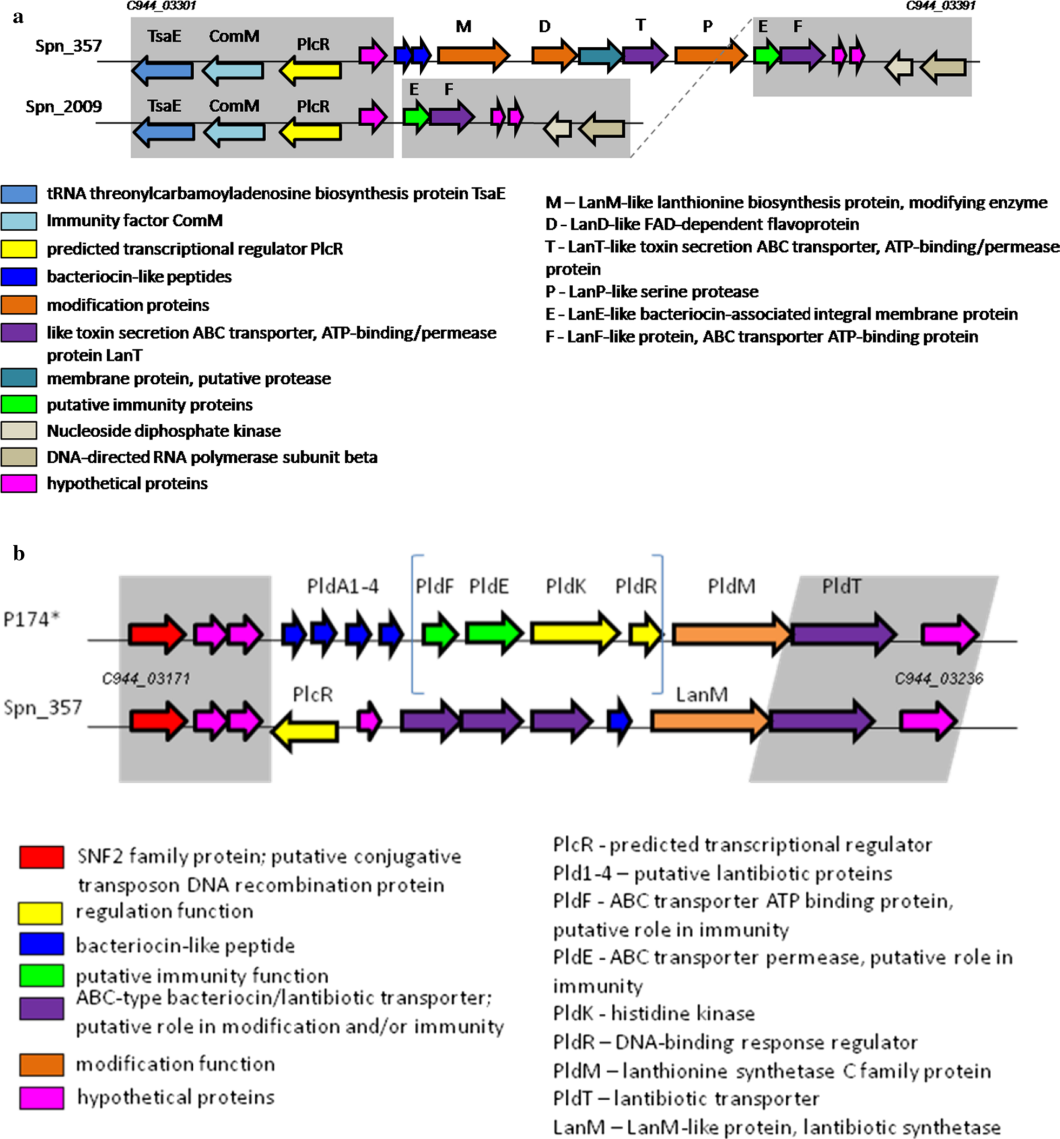
Graphic representation of lantibiotic clusters I (**a**) and *pld* locus (**b**) based on the genome analysis of strains under study. *P174 is the strain described in the work (Maricic et al. 2016) where an unusual tandem of four lantibiotic-like genes was found out. Square brackets point a part of the *pld* locus found in *S. mitis* strains cluster IV gene locus (see text). Grey fields highlight homologous fragments in different genomes

Besides of the class II bacteriocins, different species of the genus *Streptococcus* may produce class I post-translationally modified peptides termed lantibiotics (Nes et al. 2007; Hakenbeck and Chhatwal 2007). In our strains, only one lantibiotic-like peptide coding gene was discovered in the genome of Spn_357: a two-peptide bacteriocin was part of gene cluster (cluster I by Majchrzykiewicz). This cluster included genes encoding putative regulation, modification, transport and immune proteins (Fig. 2a). This cluster was missed in other genomes under study. In Spn_2009, a truncated variant of this cluster was found: modifying and transport genes were lost including bacteriocin genes.

One more pneumococcal lantibiotic locus (the pneumolancidin, *pld,* locus) has been described very recently by Maricic et al. (2016). It is located on a mobile element that has been found in some pneumococcal lineages. A special feature of *pld* locus is unusual tandem array of four inhibitory peptides, three of which are absolutely required for antibacterial activity (see Fig. 2b). An alternative variant of the lantibiotic locus that was described for *S. pneumoniae* ATCC 700669 strain (Maricic et al. 2016) includes only one lantibiotic precursor peptide (Fig. 2b). In our strains, the *pld* locus was found in Spn_357 only having an “ATCC 700669-like” structure (Fig. 2b). We were not able to detect this locus in genomes of other study strains; however, a part of it namely *pldFEKR* fragment was found in the nearest vicinity of cluster IV in two of three *S. mitis* strains (see below).

#### Lactococcin 972-like peptides in genomes of strains under study

**Fig. 3:**
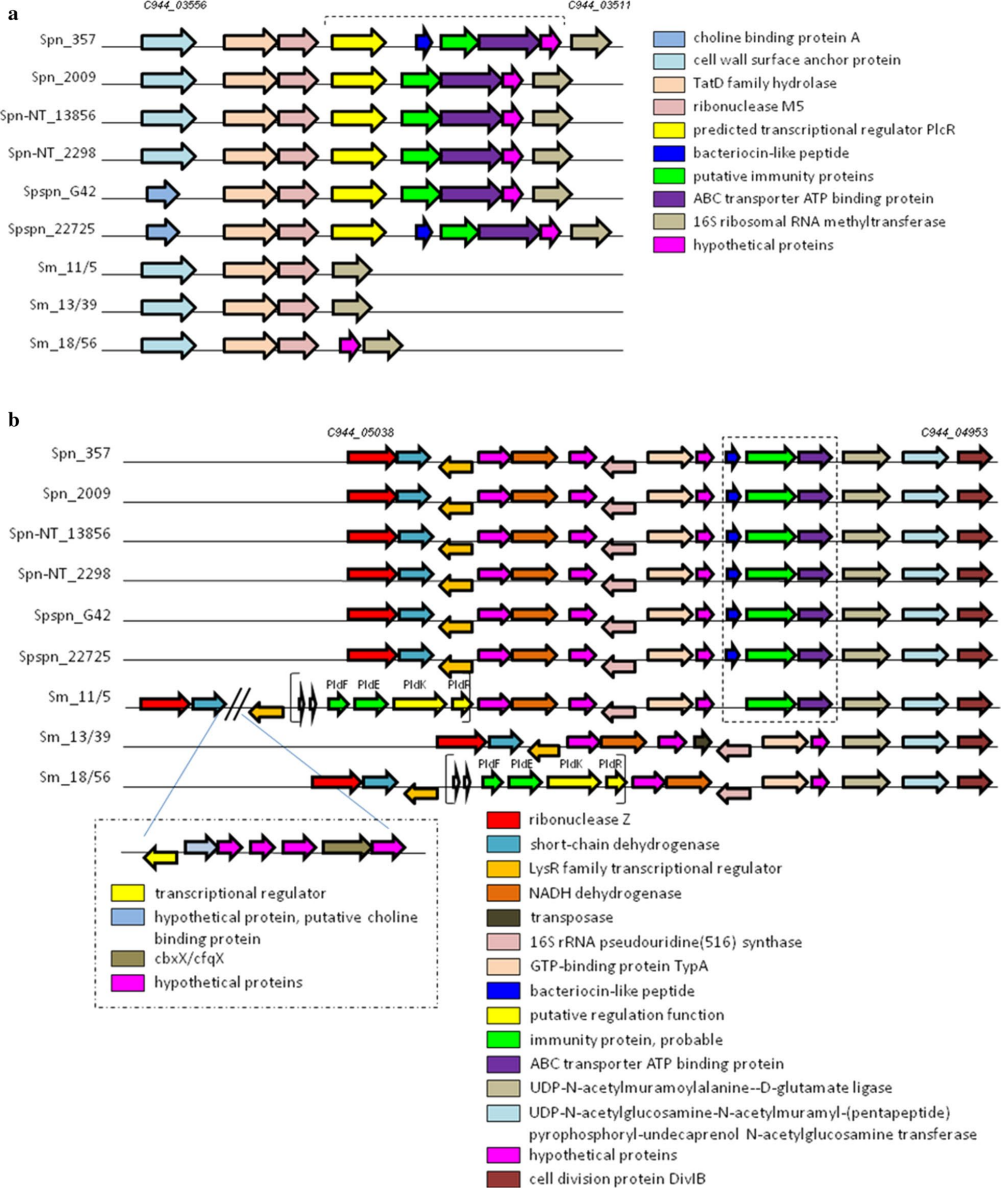
Graphic representation of lactococcin 972 clusters III (**a**) and IV (**b**), respectively. Homologous genes in different samples are indicated by the same color. Square brackets point a part of the *pld* locus in *S. mitis* genomes in the bottom picture. Unfilled arrows mark the fragments of nucleotide sequences which are homologous to *pld1*-*3* or *pld4* genes (see text)

Two lactococcin 972-like peptides were detected by BAGEL3 in our strains. Lactococcin 972 is a IIc class bacteriocin obtained from *Lactococcus lactis* that affects a target cell inhibiting cell division by blocking of septum formation (Alvarez-Sieiro et al. 2016). The corresponding loci in pneumococcal genomes were designated as clusters III and IV by Majchrzykiewicz (2011). Both clusters carry homologous genes, but they are localized in different regions of genomes. Both clusters include a bacteriocin gene, a putative self-immunity protein and ABC transporter downstream. Lactococcin-like genes were discovered within the cluster III in Spn_357 and Spspn_22725, and within the cluster IV in all genomes except *S. mitis* ones (Fig. 3). In two *S. mitis* genomes, the entire cluster was lost, whereas in Sm_11/5 immune and transport genes were preserved, but a structural gene was missed.

It should be noted that upstream of the position of the cluster IV in pneumococcal genomes, in genomes of two of three *S. mitis* strains we detected fragments of the lantibiotic *pld* operon including genes required for pneumolancidin immunity and regulation (*pldFEKR*) (Fig. 3b). In accordance with the arrangement of genes in the *pld* locus, we would expect to find pneumolancidin *pldA1*-*4* genes upstream of *pldF*. Actually, the nucleotide sequence in this region included two fragments similar to *pldA1*-*3* and *pldA4* genes. However, both nucleotide fragments were disrupted by stop codons.

#### Putative bacteriocin-coding clusters in streptococcal genomes

Inspired by results of Majchrzykiewicz, we examined two more gene loci presumably related to the bacteriocin production activity. Schemes of these loci are given in Additional file 1: Figure S1. One of them, cluster V or *ppu* (“pneumococcal peptide of unknown function), was thoroughly studied by Majchrzykiewicz, to understand whether it produces a functional bacteriocin-like peptide, but no antimicrobial activity specifically related to the PpuA bacteriocin-like peptide was revealed. We discovered the *ppuA* gene in four of nine genomes including pneumococci and pseudopneumococci, but not *S. mitis*. Other locus (cluster VI by Majchrzykiewicz) comprised of four genes encoding small peptides, putative bacteriocins. However, the function of these peptides as well as bacteriocin-like potential of the whole cluster is still unclear.

#### Sactipeptide locus in the genome of *S. mitis* 13/39 strain

**Fig. 4:**
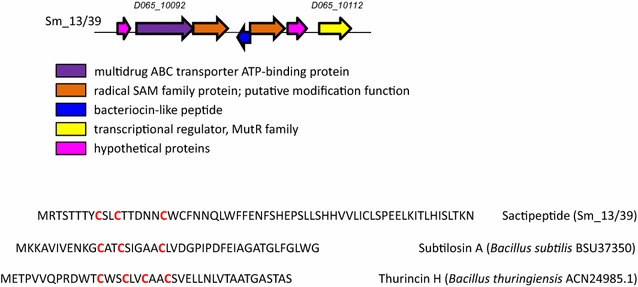
Sactipeptide cluster structure on the data of Sm_13/39 genome analysis. In the bottom of picture, the AA sequence of putative bacteriocin discovered in the Sm_13/39 genome is given compared with known sactipeptides, subtilosin A and thurincin H

One more bacteriocin-like peptide encoding gene was discovered in the Sm_13/39 genome, which was attributed to the sactipeptides. Sactipeptides represent a subclass of sulfur-bridged bacteriocins which are characterized by a typical pattern comprising three or four cysteine residues separated by a certain number of amino acids (Fig. 4). These cysteine residues serve to form intramolecular thioether bridges between cysteine sulfurs and α-carbons of other amino acids within a peptide (Himes et al. 2016). Upstream and downstream of the putative bacteriocin, two radical SAM/SPASM domain-containing proteins are located that presumably mediate post-translational thioether linkage formation (Lohans and Vederas 2014).

In the remaining studied strains, neither sactipeptide bacteriocin nor adjacent radical SAM enzymes encoding genes were detected.

#### The “*S. mitis*” bacteriocin-encoding cluster in streptococcal genomes

**Fig. 5:**
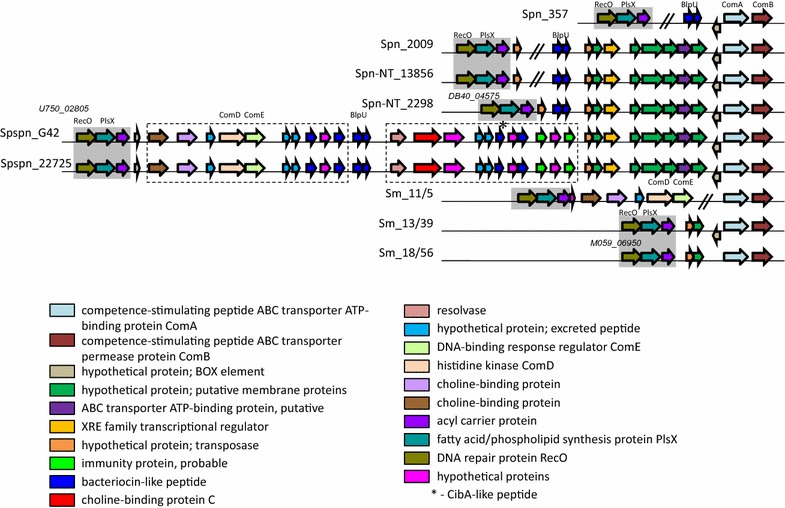
Reconstruction of the “*S. mitis*” gene cluster structure. Homologous genes in compared samples are indicated by the same color. Grey fields highlight homologous fragments in different genomes. Double slash indicates the gaps in the nucleotide sequence (a point of joining of two contigs). NCBI identifiers of the first gene in locus are given for Spn-NT_2298, Spspn_G42 and Sm_18/56, respectively

This locus upstream of *comAB* was first mentioned in the paper concerning with the analysis of the *S. mitis* B6 genome (11), so we quoted it as “*S. mitis*” cluster. Later it was also mentioned when describing the *S. pseudopneumoniae* IS7493 genome (Shahinas et al. 2013). This obscure locus seems to be very variable in different members of the *Streptococcus* genus. In our pneumococcal strains, it included genes encoding BlpU (BlpO)-like bacteriocin and (except Spn_357) a number of putative membrane proteins of unclear function (Fig. 5). Transcriptional regulator and multidrug transporter encoding genes in this locus of pneumococci and pseudopneumococci were found. Also, a BOX element directly upstream of *comAB* was conserved in all species excluding two of three *S. mitis* strains.

In two *S. pseudopneumoniae*, the arrangement of the “*S. mitis*” cluster seems to be the most interesting. First, it included a fragment of the competence regulon, namely a ComDE-like two-component regulatory system. This system plays a role at the initial stage of competence, when extracellular competence stimulating peptide pheromone (CSP) encoded by the *comC* gene is sensed by histidine kinase receptor ComD, which, upon binding of its ligand, transfers a phosphoryl group to the response regulator ComE. Phosphorylated ComE drives the expression of early competence genes (Claverys and Havarstein 2007). However, we revealed no homologous of *comC* gene in the vicinity of *comDE*. Note that we found a competence regulated *cibA*-like gene at this locus in both pseudopneumococci strains. At the same time, there is a full repertoire of genes of the competence regulon located at the position equivalent to that of the *S. pneumoniae* genome in both *S. pseudopneumoniae* strains.

Second, in addition to BlpU, a large number of the putative class II bacteriocin-like peptide encoding genes with a typical GG-processing site were present in this locus. Also, two immune protein encoding genes upstream of the field of putative membrane proteins were discovered. Finally, a few of excreted peptides of unknown function were localized there.

Surprisingly, “*S. mitis*” cluster was completely lost in two of three *S. mitis* genomes and truncated in the third one (Sm_11/5) that preserved only the regulatory *comDE* part.

## Discussion

In this study, we investigated an ability of *S. pneumoniae* and its closest commensal relatives—*S. pseudopneumoniae* and *S. mitis*—to inhibit the growth of *M. catarrhalis* strains. *Moraxella catarrhalis* have been chosen as indicator strain because it shares the same niche as viridans group streptococci—upper respiratory tract—in a human body (Bosch et al. 2013; Perez et al. 2014), so we might expect a manifestation of established competitive relationships between these species.

Our experiments show some important features. First, VGS streptococci are able to suppress the growth of other microorganism, at that this process is probably mediated substantially by the production of hydrogen peroxide which is inherent for this genus. However, an inhibiting ability is partially kept in all the strains under study even after inactivation of hydrogen peroxide by catalase. At that, the inhibitory effect of both pneumococci and pseudopneumococci on the *M. catarrhalis* strains growth should be probably attributed to the living cells of microorganisms, because of the treatment in chloroform vapors leads to the disappearance of this effect. On the contrary, the inhibiting ability of *S. mitis* strains does not disappear when the bacterial cells are killed in chloroform.

We suggested that this inhibitory effect could be associated with the production of bacterial antimicrobial peptides, so we scanned the genomes of our strains for the presence of appropriate mechanisms for bacteriocins production. Note that this work was not aimed the isolation and characterization of an inhibitory substance, as well as a systematization of all diversity of bacteriocins from streptococcal genomes available from the online databases. We just assumed to identify a basis of the inhibitory ability of strains under study which we observed in the experiments. Also, it seemed interesting to compare “the potential of competitiveness” of pathogen *S. pneumoniae* and commensals *S. pseudopneumoniae* and *S. mitis*, because the ability of each of these species to survive inside a competitive microbial community affects the clinically important nasopharyngeal dynamics.

The results that we obtained were rather unexpected. We discovered many opportunities of pneumococci and pseudopneumococci to produce bacteriocins, and first of all it was associated with the presence of the *blp* locus which was found intact in all *S. pneumoniae* and *S. pseudopneumoniae*. In addition to the *blp* operon, there were a number of other loci in the genomes of *S. pneumoniae* and *S. pseudopneumoniae* which can be associated with a possibility to produce bacteriocins. We can only guess whether all these loci are joined into the complex system, and if so, how this system works, or maybe it is only partially or completely dysfunctional gene clusters that are nothing more than the evolutionary heritage. In any case, all that ‘potential of bacteriocinogeny”, it seems, was not used by these bacteria in the conditions of our experiment, since the inhibitory effect completely disappeared when the cells were killed in chloroform vapors.

But the most intriguing observation was the finding of significant inhibitory ability of *S. mitis* strains, which was kept even after killing of the test strains in chloroform, in conjunction with a virtually complete lack of bacteriocin-like peptide encoding genes in their genomes. Actually, the “potential of bacteriocinogeny” of *S. mitis* strains involved into this work inferred on the basis of the analysis of their genomes looks quite poor in comparison with pneumococci and pseudopneumococci. We succeeded to detect two bacteriocin-like peptide encoding genes inside of the *blp* and *cibAB* loci of *S. mitis* Sm_11/5 strain and only one sactipeptide-like encoding gene in the Sm_13/39 strain. No bacteriocin encoding genes were discovered in Sm_18/56 genome. A thorough study of loci, which are presumably associated with bacteriocin production activity in *S. pneumoniae*, did not clarify the situation with this strain. The significant inhibitory potential of alpha-hemolytic streptococci including *S. mitis* against a variety of Gram-positive and Gram-negative bacteria was observed earlier. The “viridin B”, a bacteriocin produced by *S. mitis* and active against *Neisseria sicca* and coagulase-negative staphylococci, was purified and described in a number of papers almost four decades ago (Law and Dajani 1978; Dajani et al. 1978), but the sequence of this peptide has remained unknown since then. This substance was obtained in a cell-free form only after mechanical disruption of bacteriocinogenic cells but has not been isolated from streptococcal culture supernatants (Dajani et al. 1976), so it’s hard to speculate what was it in fact. Much later, a broad inhibitory activity of *S. mitis* strains against different microorganisms including *S. pyogenes*, *S. pneumoniae, S. aureus,* and *B. catarrhalis* has been demonstrated again (Santagati et al. 2012). A targeted search for the known streptococcal bacteriocin genes resulted in finding of *salA* or *sboB* (encoding lantipeptides salivaricin A or B, respectively) genes in some strains; however, other isolates showing an evident inhibition of the indicator strains did not possess any of these bacteriocin activity determinants.

Thus, the origin of inhibition activity of at least one *S. mitis* strain remains unclear. Perhaps, there is some novel type of antimicrobial peptides in them that has not yet been discovered, or some secreted metabolites which are remained to be identified. In any case, we can see that commensals *S. mitis* are able to compete effectively for their place in the conditions of our experiment, and their competitive tools seem to be different from those of pneumococci.

## Additional file

**Additional file 1: Table S1.** Details of whole genome sequencing of streptococci strains under study. **Figure S1**. Graphic representation of putative bacteriocin-associated clusters V and VI.

## Abbreviations

NT: non-typeable pneumococci
OPT: optochin
MLSA: multilocus sequence analysis
MLST: multilocus sequence typing
ST: sequence type
