# Supplementary material for: Inhibitory effect of streptococci on the growth of M. catarrhalis strains and the diversity of putative bacteriocin-like gene loci in the genomes of S. pneumoniae and its relatives
Source: AMB Express. 2017 Dec 13;7:218. doi: 10.1186/s13568-017-0521-z (PMC5729180; doi:10.1186/s13568-017-0521-z)
Supplement: Supplementary file 1 — Additional file 1: Table S1. Details of whole genome sequencing of streptococci strains under study. Figure S1. Graphic representation of putative bacteriocin-associated clusters V and VI. [file 13568_2017_521_MOESM1_ESM.pdf]

*Journal name*: AMB Express

*Manuscript Title*: Inhibitory effect of streptococci on the growth of *M. catarrhalis* strains and the diversity of putative bacteriocin-like gene loci in the genomes of *S. pneumoniae* and its relatives

*Authors*: L.N. Ikryannikova, M.V. Malakhova, G.G. Lominadze, I.Yu. Karpova, E.S. Kostryukova, N.A. Mayansky, A. N. Kruglov, E.A. Klimova, E.S.Lisitsina, E.N. Ilina, and V.M. Govorun

*Affiliations*:

L.N. Ikryannikova, M.V. Malakhova, I.Yu. Karpova, E.S. Kostryukova, E.N. Ilina, V.M. Govorun - Federal Research and Clinical Center of Physical-Chemical Medicine, 119992 Malaya Pirogovskaya str., 1a, Moscow, Russia;

G.G. Lominadze - Federal State Budgetary Inst. "Scientific Center of Children Health" of RAMS, 119991, Lomonosovsky prospekt, 2, b.1, Moscow, Russia;

N.A. Mayansky - I.M. Sechenov First Moscow State Medical University, 119991, 2-4 Bolshaya Pirogovskaya st., Moscow, Russia; Federal State Budgetary Inst. "Scientific Center of Children Health" of RAMS, Moscow, Russia;

A. N. Kruglov - National Agency for Clinical Pharmacology and Pharmacy, 115088, Ugreshskaya str., 2, b. 8, Moscow, Russia;

E.A. Klimova - A.I. Evdokimov Moscow State University of Medicine and Dentistry, 127473, Delegatskaya str., 20, b. 1, Moscow, Russia.

E.S. Lisitsina - [6]LTD Scientific and Industrial Company "Lytech", Moscow, Russia

*Corresponding author*: L.N. Ikryannikova. Federal Research and Clinical Center of Physical-Chemical Medicine. 119992 Malaya Pirogovskaya str., 1a, Moscow, Russia. Tel/fax: +7(499)246-45-70, 246-45-01 E-mail: Larisa.Ikryannikova@gmail.com

Table S1. Details of whole genome sequencing of viridans group streptococci under study.

| Strain | Sequencing Platform | Sequencing Depth | Number of Large Contigs | Genome Length | Accession number |
|---|---|---|---|---|---|
| **Spn_357** | Ion Torrent PGM™ | 35x | 115 | 2098179 | GCA_000385775.1 |
| **Spn_2009** | Ion Torrent PGM™ | 34x | 103 | 2020583 | GCA_000385795.1 |
| **Spn-NT_13856** | Ion Torrent PGM™ | 43x | 120 | 2111028 | GCA_000506605.2 |
| **Spn-NT_2298** | GS FLX+ | 17x | 117 | 2181986 | GCA_000692035.1 |
| **Spspn_G42** | Ion Torrent PGM™ | 108x | 146 | 2118314 | GCA_000506665.1 |
| **Spspn_22725** | GS FLX+ | 18x | 93 | 2223262 | GCA_000506685.1 |
| **Sm_11/5** | Ion Torrent PGM™ | 75x | 60 | 1872648 | GCA_000385815.1 |
| **Sm_13/39** | Ion Torrent PGM™ | 48x | 110 | 2045294 | GCA_000385835.1 |
| **Sm_18/56** | Ion Torrent PGM™ | 53x | 67 | 1917104 | GCA_000430345.1 |

**A**

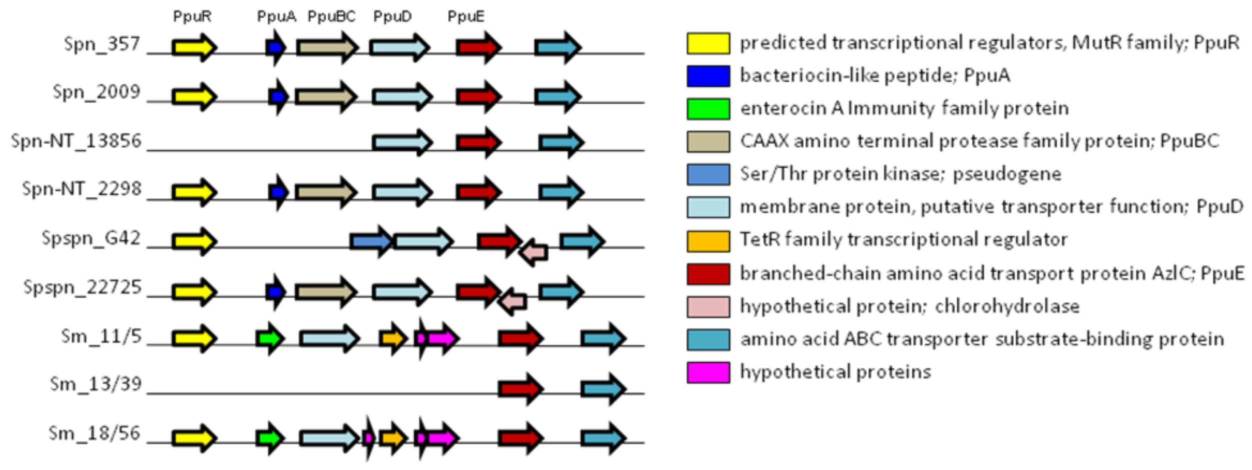


**B**

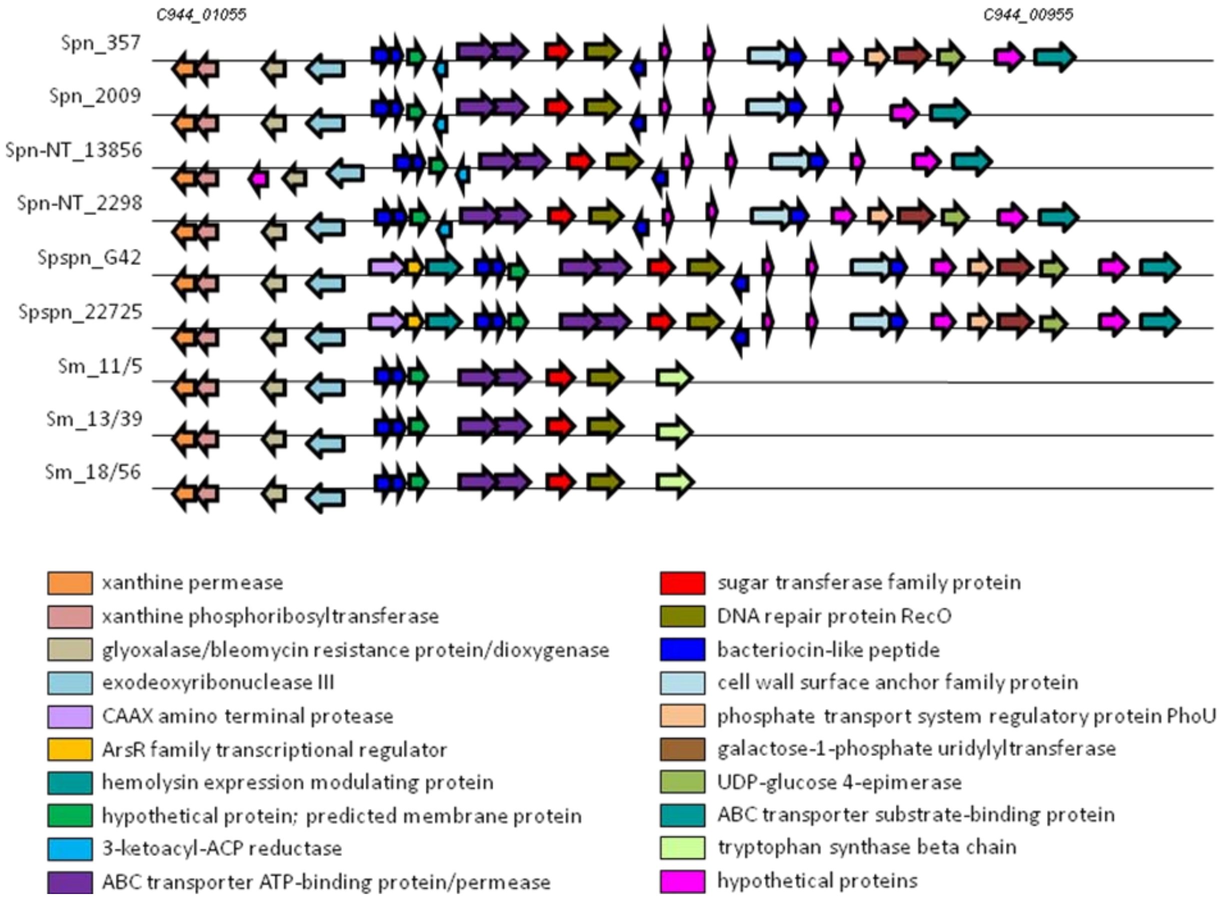


Figure S1. Graphic representation of putative bacteriocin-associated cluster V, or *ppu* (**A**) and cluster VI (**B**), respectively (see (Majchrzykiewicz JA. Bacteriocins of Streptococcus pneumoniae and its response to challenges by antimicrobial peptides. Groningen: s.n., 2011. 174 p.)). Homologous genes

in different samples are indicated by the same color. NCBI identifiers of the first and last genes of genome fragments presented are given for Spn_357.
